# Risk Factors for Early Postoperative Morbidity and Mortality following Extremity Metastatic Pathologic or Impending Fracture Fixation

**DOI:** 10.1155/2024/3565134

**Published:** 2024-09-12

**Authors:** Patrick Qi Wang, Brynn P. Charron, Kalter Hali, Matthew Raleigh, Christopher Del Balso, Mark D. Macleod, David W. Sanders, Abdel-Rahman Lawendy

**Affiliations:** ^1^ Western University, London, Ontario, Canada; ^2^ University of Toronto, Toronto, Ontario, Canada

## Abstract

**Background:**

As cancer survivorship continues to improve, the perioperative morbidity and mortality following surgical treatment of metastatic bone disease become an increasingly important consideration. The objective of this study is to identify risk factors for early postoperative complications and mortality following extremity prophylactic fixation and pathologic fracture stabilization.

**Methods:**

A single-centre retrospective review of 185 patients (226 total surgeries) who underwent prophylactic fixation or pathologic fracture stabilization for extremity metastatic bone disease between 2005 and 2020 was performed. Skull, spine, pelvic, and revision surgeries along with diagnosis of primary bone neoplasm were excluded. Univariate, multivariate, and subgroup analyses were performed to identify predictors and independent risk factors for 30-day postoperative morbidity and mortality.

**Results:**

Primary cancers included lung (*n* = 41), breast (*n* = 36), multiple myeloma (*n* = 35), prostate (*n* = 16), lymphoma (*n* = 11), renal cell carcinoma (*n* = 10), and *other* (*n* = 36). The 30-day postoperative complication and mortality rates were 32.30% (*n* = 73) and 17.26% (*n* = 39), respectively. The most common complications were pulmonary-related, cardiac events, surgical site infection, sepsis, and thromboembolism. Pathologic fracture, presence of extra-skeletal metastases, longer surgical duration, and blood transfusion requirements were associated with 30-day postoperative complications overall. A past medical history for cardiac disease was also associated with systemic but not local surgical complications. Pathologic fracture, presence of extra-skeletal metastases, lung cancer, blood transfusion requirements, and increasing pack-year smoking history were predictors for 30-day mortality. In the multivariate analysis, pathologic fracture (*p*=0.016) and presence of extra-skeletal metastases (*p*=0.029) were independent predictors of complications. For mortality, pathologic fracture (*p*=0.014), presence of extra-skeletal metastases (*p*=0.0085), and increased blood transfusion requirements (*p*=0.048) were independent risk factors.

**Conclusions:**

The findings of this study provide additional guidance for perioperative risk assessment and patient counselling. Additionally, improving clinical assessment tools to identify and quantify patients at risk of pathologic fractures becomes increasingly important given the significant morbidity and mortality associated with pathologic fracture treatment.

## 1. Introduction

The incidence of metastatic bone disease is expected to continue to rise as the life expectancy of cancer patients continues to increase resulting from advances in screening techniques as well as local and systemic treatments. However, despite the progress in cancer care, reaching the stage of metastasis generally signifies irreversible disease course [1–4]. Currently, up to 80% of patients with advanced disease will experience bone metastases and 10–29% will suffer from pathologic fracture(s) [1, 5–7]. Unfortunately, these skeletal events carry significant consequences with debilitating pain, loss of independence, impaired quality of life, and decreased survival time, in addition to the emotional burden for patients and family members [1, 3, 8, 9].

The reported one-year survival rate following a pathologic fracture is between 30% and 70%, while the 2-year survival rate is less than 30%, all cancer aetiologies included [2, 6, 10–14]. Therefore, the aim of surgical intervention is immediate pain relief, restoration of function, and improved quality of life often in the setting of end-of-life care, while minimizing perioperative complications [1, 3, 5, 14–16]. At present, increased emphasis has been placed on creating models to predict symptomatic skeletal events from the time of cancer diagnosis and to identify prognostic factors for overall survival. However, there are limited data exploring the risk factors for early postoperative morbidity and mortality from the time of impending or pathologic fracture diagnosis, which is usually when patients are seen by an orthopaedic surgeon for the first time. This has great implications in clinical decision-making, perioperative risk assessment, as well as patient and family counselling [1, 6, 8, 12, 17, 18].

The objective of this study is to identify risk factors for short-term complications and mortality in the surgical treatment of extremity metastatic bone disease. Ultimately, the aim is to provide decision-making guidance and to enhance perioperative risk assessment.

## 2. Methods

A retrospective review of consecutive patients who underwent surgical treatment for impending and/or pathologic fractures related to extremity metastatic bone disease from January 1, 2005, to December 31, 2020, was performed. Primary bone neoplasm, revision surgeries, nonsurgical management, and treatment of skull, spine, pelvis, or nonskeletal metastatic disease were excluded. All patient consultation and surgical interventions were performed at a single tertiary centre with regional cancer care designations.

The data were independently collected by two authors (PQW, BPC) from the institution's electronic medical record. Discrepancies were reviewed until consensus was reached. Primary endpoints were 30-day postoperative complications and mortality. A complication was defined as a new or worsening condition requiring additional medical or surgical treatment. An impending fracture was defined as a metastatic bone lesion without a visible fracture line, with intact height and bone alignment, and which underwent prophylactic fixation [19]. For risk factor assessment, variables of interest included age, sex, primary cancer, time (in months) between cancer diagnosis and surgery, chemotherapy and radiation therapy history, fracture location, prophylactic versus fracture stabilization, type of fixation, type of anesthesia, surgical duration, metastatic burden, medical comorbidities, smoking history, preoperative (or prefracture) ambulatory status, and transfusion requirements. Metastatic burden was dichotomized into either bone metastasis alone or presence of extra-skeletal metastases. Surgical location was categorized as upper extremity, lower extremity, and multiple limbs given the propensity to metastasize to both the humerus and femur, and less frequently to other bones [12, 19]. Medical comorbidities needed to be preexisting conditions and unrelated to the cancer diagnosis and/or treatment. The smoking history and blood transfusion requirements were assessed using categorical values (yes/no) as well as continuous variables (pack-year smoking history and number of blood product units transfused, respectively). Where patients underwent multiple separate surgeries for different limbs within 30 days and sustained a complication or incurred death, these data were linked only to the most recent surgery and not the previous interventions as the patient would have been expected to be optimized for surgery.

Patient demographics, clinical characteristics, 30-day morbidity, and mortality were summarized using descriptive statistics. Categorical variables were presented as frequencies and percentages, whereas continuous independent variables were reported as mean, standard deviation, and range. Postoperative morbidity and mortality were assessed separately using univariate and multivariate analyses. Associations between each variable and outcome were identified using chi-squared or Fischer's exact test for categorical values. A binary logistic regression model was used for continuous independent variables. A multivariate regression analysis was subsequently performed to identify independent risk factors for postoperative complications and mortality. Variables that were statistically significant in the univariate analysis were included in the multivariate model and were reported as odds ratio (OR) with 95% confidence interval (CI). An additional subgroup analysis was performed to identify specific risk factors for local surgical and systemic complications separately. Complications occurring outside the surgical field were considered systemic. Statistical significance was set at *p* value less than 0.05. Statistical analysis was performed using GraphPad Prism 10 (San Diego, California, USA).

Approval from the institution's research ethics committee was obtained prior to initiating data collection. This study followed the *Strengthening the Reporting of Observational Studies in Epidemiology* (STROBE) guidelines [20].

## 3. Results

Of the 185 patients included in the study, 30 underwent multiple surgeries on separate occasions for different extremities leading to a total of 226 surgeries. See Table 1 for patient demographics and clinical characteristics and Table 2 for surgical details. The most common primary cancer was lung cancer (*n* = 41) followed by breast cancer (*n* = 36), multiple myeloma (*n* = 35), prostate cancer (*n* = 16), lymphoma (*n* = 11), and renal cell carcinoma (*n* = 10). The remaining cancers were combined as *other* (*n* = 36) as the number of each individual pathology was insufficient for statistical analysis, see Table 1. Fifty patients (27.03%) only had skeletal metastatic lesions at the time of surgery, while 135 (72.97%) had both bone and extra-skeletal metastases.

The 30-day postoperative complication and mortality rates were 32.30% (*n* = 73) and 17.26% (*n* = 39), respectively. The most common morbidity was respiratory-related complications (*n* = 26). Amongst them, 15 sustained new or worsening pleural effusions, six were treated for pneumonia, and five had new onset of pulmonary embolism. Other complications observed include superficial wound infection (*n* = 8), cardiac event (*n* = 7), deep surgical infection (*n* = 6), urosepsis (*n* = 6), deep vein thrombosis without pulmonary embolism (*n* = 4), gastrointestinal bleed (*n* = 3), failure of fixation (*n* = 3), acute renal failure (*n* = 3), symptomatic hyponatremia (*n* = 2), *Clostridioides difficile* infection (*n* = 1), febrile neutropenia (*n* = 1), hypercalcemia of malignancy (*n* = 1), seizure (*n* = 1), and hepatic encephalopathy (*n* = 1).

Univariate analysis showed that pathologic fracture (*p*=0.0064), presence of extra-skeletal metastases (*p*=0.046), longer surgical duration (*p*=0.021), and blood transfusion (yes/no, *p*=0.03) were associated with 30-day postoperative complications. Predictors for 30-day mortality were lung cancer (*p*=0.046), pathologic fracture (*p*=0.0071), presence of extra-skeletal metastases (*p*=0.022), and blood transfusion (yes/no, *p*=0.031). When patients received increasing amounts of blood transfusion units, this also significantly influenced both morbidity and mortality (*p*=0.045 and *p*=0.015, respectively). Although smoking status (ever-smoking versus nonsmoker) was not correlated with mortality, the pack-year smoking history significantly affected the deceased group (*p*=0.033), see Tables 3 and 4.

A multiple logistic regression analysis of statistically significant factors on univariate analysis was performed. The model showed pathologic fracture (OR: 2.14, 95% CI: 1.16 to 4.00, *p*=0.016) and presence of extra-skeletal metastases (OR: 2.12, 95% CI: 1.14 to 4.37, *p*=0.029) as independent predictors for 30-day postoperative complications. For 30-day mortality, independent risk factors were pathologic fracture (OR: 2.84, 95% CI: 1.27 to 6.86, *p*=0.014), presence of extra-skeletal metastases (OR: 4.14, 95% CI: 1.56 to 13.44, *p*=0.0085), and increasing amounts of blood transfusion units required (OR: 1.27, 95% CI: 1.00 to 1.62, *p*=0.048), see Table 5.

The subgroup analysis assessing risk factors stratified by local (*n* = 17) and systemic (*n* = 56) complications showed that pathologic fracture (*p*=0.012), presence of extra-skeletal metastases (*p*=0.0051), cardiac medical history (*p*=0.049), and increasing amounts of blood transfusion units (*p*=0.032) were associated with systemic complications. However, no statistically significant risk factors were identified for local surgical complications alone.

## 4. Discussion

The objective of this study was to identify the risk factors for short-term postoperative morbidity and mortality to guide clinical decision-making and enhance perioperative risk assessment in the treatment of impending and pathologic fractures of the extremity due to metastatic bone disease. Several risk factors were identified, of which pathologic fractures, presence of extra-skeletal metastases, and increased blood product requirements were independently associated with morbidity and/or mortality. A cardiac past medical history was also a significant risk factor for systemic complications in the subgroup analysis. The most common complications included pulmonary-related morbidity, cardiac events, surgical site infections, sepsis, and thromboembolism.

The treatment of pathologic fractures, similarly reported in previous studies, has significantly worse outcomes compared to prophylactic stabilization and is an independent negative prognostic factor in the treatment of extremity metastatic disease [11, 12, 21, 22]. Previous studies demonstrated that long bone pathologic fracture fixation compared to prophylactic stabilization resulted in a significantly higher rate of 30-day major medical complications, longer hospital stays, and higher likelihood of patient discharge into a secondary health care facility rather than home discharge [19, 23] and is associated with an increased 1-year mortality rate [19, 24]. Consequently, identifying patients at a higher risk of pathologic fracture and advocating for treatment of impending rather than completed fractures, all factors considered, is becoming increasingly important. At present, assessment tools quantifying true risks of pathologic fractures are still limited. Mirel's scoring system is still the most widely used screening tool for predicting the risk of pathologic fracture. However, its low specificity rate (35%) leads to a high false-positive rate, thereby leading to overtreatment of extremity metastatic bone disease if decisions were made solely based on Mirel's score. Additionally, previous studies have demonstrated a moderate interobserver agreement only. Computed tomography-based bone assessment models have shown promising results in predicting the risks of pathologic fractures. However, given its complexity, it is still limited in availability and lacks standardization [22, 23, 25–28].

Prognostic predictive models such as PATHFx, 2013-SPRING, Optimodel, and SORG (the Skeletal Oncology Research Group) have been developed over the years to assess overall survival prognosis following treatment of metastatic bone disease. However, they do not quantify the risk of completed pathologic fracture. While helpful, these clinical algorithms have not been validated as stand-alone decision-making tools for management of patients with metastatic bone disease, rather, a multidisciplinary approach remains the gold standard. Furthermore, these tools are not yet used routinely, even amongst orthopaedic oncologists. As a result, emphasis should be placed on developing precise and accurate clinical prognostic tools both in quantifying risks of pathologic fracture as well as prognosis to make better informed decisions. These tools should be simple to apply and available, not only for clinicians in tertiary oncology referral centres, but also for secondary centres who also treat these patients. This is ever-more important now that patients survive longer and an increasing number of symptomatic skeletal events are expected to occur [22, 26, 29–32].

Similar to this study, reported acute postoperative complications and mortality are observed in up to 31% and 19%, respectively [12, 19, 21, 29]. The most common reported postoperative complications are respiratory complications, cardiac events, surgical site infections, sepsis, and thromboembolism [18, 19, 23]. Common prognostic factors for morbidity and/or mortality previously published include rapidly growing primary tumours, lung cancer, disseminated disease, age (older), blood transfusion requirements, lower extremity location, and several laboratory values [18, 19, 29]. Unlike previous articles, in the current study, tumours were not differentiated by slow, moderate, and rapid growth, although lung cancer, known for its aggressive potential, was a risk factor in the univariate analysis for mortality, and metastatic burden (advanced disease) was an independent factor for both morbidity and mortality. Laboratory values were categorized as a complication only if they required additional treatment, thus accounting for the discrepancies from previous data. In addition, perioperative albumin levels were not routinely performed. It has been postulated that blood transfusion alone may not be a prognostic factor, rather it is the low preoperative hemoglobin level and perioperative anemia that affect patient outcomes, hence potentially why transfusion requirement (yes/no) was not an independent risk factor in this study, but mortality was affected by the increasing amount of blood transfusion requirements [11, 18]. A cardiac medical history as a risk factor for systemic complications was identified in the subgroup analysis but not in the univariate analysis, although it did trend towards, but did not reach significance. Thus, cardiac disease likely has a certain effect on patient morbidity, and the discrepancy could be explained by the low sample size and heterogeneity in data.

This study did not show an increased risk of morbidity for lower extremity compared to upper extremity surgery, unlike observations made from previous studies. First, this could be explained by differences in patient demographics, cancer status, type of surgery, and how complications were defined. Additionally, lower extremity surgeries often require longer hospital stays and more intense rehabilitation compared to the upper extremity. However, most studies assess long-term morbidity, likewise with implant-related failures and complications. Consequently, morbidity related to lower extremity surgery and implant failures likely become more significant beyond the initial 30-day period [19, 33–36].

There is conflicting evidence as to whether older age is an independent risk factor in long-term outcome studies. Previous pooled analyses proposed some studies showing age as an independent risk factor, while other studies did not reproduce the same results [11]. Differences can be influenced by the cancer types and whether they are assessed separately or together. For example, age affects survival in breast, prostate, renal cell, and thyroid cancers when analyzed independently [11, 37–41]. However, studies with pooled primary cancer diagnoses, often do not demonstrate age as a prognostic factor [12, 42].

The current study did not demonstrate associations between prior chemotherapy and prior radiation with short-term morbidity or mortality. Similarly, Bindels [19] did not find correlations between 30-day complications and previous systemic therapies or radiation to the affected bone [19]. However, given that disease dissemination and metastatic burden affect outcomes, it is more likely that the actual response to systemic treatment will have a greater impact on overall survival, especially if it can delay disease progression [7, 43].

This study has several limitations stemming from its retrospective design, heterogenous patient demographics and characteristics, and its reliance on electronic medical record. With a relatively small sample size, not all variables could be independently analyzed. As a result, cancer types and complications were pooled together instead to increase power. Furthermore, in the literature, there is no uniform definition and data collection method for complications, thereby making direct comparisons difficult [19]. Certain types of surgical procedures from different locations were also combined to increase subgroup sample size, notably for joint reconstruction surgeries. Total joint replacements and megaprosthetic reconstructions were pooled with hemiarthroplasties of the shoulders, hips, and knees although their risk profiles differ, thereby increasing the risk of bias. Other potential confounding factors such as nutritional status, albumin levels, and various laboratory bloodwork could not be independently analyzed due to lack of data. Rather, only the reported abnormal laboratory values that required additional treatment were categorized as a complication. Additionally, information regarding oncology-related patient performance status, an important prognostic factor for survival, was not available in the chart review. Therefore, this study assessed ambulatory status as a surrogate measure. While preoperative ambulatory status did not appear to affect the short-term results, ambulatory status alone is not an adequate representation of functional status [29].

## 5. Conclusions

This study highlights the various complications and risk factors associated with early postoperative morbidity and mortality following surgery for extremity metastatic bone disease. After adjusting for potential confounders, pathologic fractures and the presence of extra-skeletal metastases were independent risk factors for morbidity and mortality. Meanwhile, increased blood transfusion requirement was a negative prognostic factor for mortality. Finally, as patient survivorship continues to improve, there is an increasing role for prophylactic treatment given their superior clinical outcomes and the morbidity associated with pathologic fracture surgery. Accurately identifying patients at a risk for pathologic fracture is paramount.

## Figures and Tables

**Table 1:** Demographic data and clinical characteristics.

Variables	Frequency (%) or mean (SD, range)
Sex^1^	
Female	94 (50.81%)
Male	91 (49.19%)
Mean age at time of surgery^2^	67.35 (11.49, 19–91)
Mean time (in months) between index cancer diagnosis and surgery^2^	33.09 (52.24, 0–358)
Primary cancer^1^	
Lung	41 (22.16%)
Breast	36 (19.46%)
Multiple myeloma	35 (18.92%)
Prostate	16 (8.65%)
Lymphoma	11 (5.95%)
Renal cell carcinoma	10 (5.41%)
Colorectal, hepatocellular	5 each (5.41%)
Carcinoma of unknown origin, melanoma, urothelial	3 each (4.86%)
Oropharynx, plasmacytoma, squamous cell carcinoma (skin), uterine	2 each (4.32%)
Endometrial, esophageal, granular cell tumour, hemangiopericytoma, maxillary sinus, merkle cell, neuroendocrine, pancreas, sarcomatoid carcinoma	1 each (4.86%)
Metastatic burden^1^	
Bone only	50 (27.03%)
Bone and extra-skeletal	135 (72.97%)
Prefracture ambulatory status^1^	
Independent	87 (47.03%)
Gait aid	52 (28.11%)
Nonambulator	27 (14.59%)
Unknown	19 (10.27%)
Medical history^1^	
Cardiac disease	39 (21.08%)
Pulmonary disease	32 (17.30%)
Vascular disease	10 (5.41%)
Diabetes mellitus	35 (18.92%)
Renal failure	13 (7.03%)
Liver disease	3 (1.62%)
Nononcologic immunosuppressive disease^1^	13 (7.03%)
Smoking history^1^	
Yes	99 (53.51%)
No	86 (46.49%)
Mean smoking pack-year history^1^	15.99 (19.93, 0–100)
Blood transfusion^2^	
Yes	91 (40.27%)
No	135 (59.73%)
Mean units of blood products transfused^2^	1.20 (1.98, 0–14)
Impending versus pathologic fracture^2^	
Impending fracture	98 (43.36%)
Pathologic fracture	128 (56.64%)
Surgical location(s) for each surgery^2^	
Femur	158 (69.91%)
Humerus	53 (23.45%)
Tibia	5 (2.21%)
Femur and humerus	4 (1.77%)
Femur bilateral	4 (1.77%)
Clavicle	1 (0.44%)
Ulna	1 (0.44%)
Type of anesthesia^2^	
General	218 (96.46%)
Neuroaxial	8 (3.54%)
Operative time^∗^ (in minutes)^2^	78 (34.30, 27–258)

^1^Data expressed according to number of patients (*n* = 185), ^2^Data expressed according to the number of surgeries (*n* = 226), SD: standard deviation, ^∗^surgery time is measured from initial incision until final wound dressing is placed.

**Table 2:** Details of surgical procedures.

Surgical intervention	Frequency (%)
For pathologic fracture	
Humerus CCP	29 (12.83%)
Femur CCP	7 (3.10%)
Tibia CCP	1 (0.44%)
Femur IMN	60 (26.55%)
Humerus IMN	6 (2.65%)
Tibia IMN	1 (0.44%)
Clavicle IMN	1 (0.44%)
Hip hemiarthroplasty	13 (5.75%)
THA	2 (0.88%)
DFR	1 (0.44%)
RTSA	1 (0.44%)
For impending fracture	
Humerus CCP	16 (7.08%)
Femur CCP	1 (0.44%)
Tibia CCP	1 (0.44%)
Ulna CCP	1 (0.44%)
Femur IMN	69 (30.53%)
Tibia IMN	2 (0.88%)
Hip hemiarthroplasty	4 (1.77%)
THA	1 (0.44%)
Proximal humerus hemiarthroplasty	1 (0.44%)
Multiple procedures in one surgery	
Bilateral femur impending fractures: Bilateral IMN	2 (0.88%)
Bilateral femur pathologic fractures: Bilateral CCP	1 (0.44%)
Bilateral femur pathologic fractures: Hip hemiarthroplasty and contralateral CCP	1 (0.44%)
Humerus and femur pathologic fractures: Humerus CCP and femur IMN	2 (0.88%)
Humerus pathologic fracture, femur impending fracture: Humerus and femur IMN	1 (0.44%)
Humerus pathologic fracture, femur impending fracture: Humerus CCP and femur IMN	1 (0.44%)

CCP: curettage, cement, plate fixation, DFR: distal femur replacement, IMN: intramedullary nail, RTSA: reverse total shoulder arthroplasty, THA: total hip arthroplasty.

**Table 3:** Univariate analysis for 30-day complications.

Variable	Frequency (%) or mean (SD, range)		*p* value
	Complication (*n* = 73)	No complication (*n* = 153)	
Sex			0.48
Female	35 (29.91%)	82 (70.09%)	
Male	38 (34.86%)	71 (65.14%)	
Mean age at time of surgery	66.63 (10.86, 42–90)	67.69 (11.80, 19–91)	0.52
Mean time (in months) between index cancer diagnosis and surgery	27.27 (46.63, 0–237)	35.86 (54.64, 0–358)	0.25
Primary cancer			0.57
Multiple myeloma	14 (26.92%)	38 (73.08%)	
Lymphoma	3 (25.00%)	9 (75.00%)	
Lung	16 (37.21%)	27 (62.79%)	
Breast	12 (26.67%)	33 (73.33%)	
Renal cell	5 (38.46%)	8 (61.54%)	
Prostate	5 (26.32%)	14 (73.68%)	
Other	18 (42.86%)	24 (57.14%)	
Metastatic burden			**0.046**
Bone only	16 (22.54%)	55 (77.46%)	
Bone and extra-skeletal^a^	57 (36.77%)	98 (63.23%)	
Previous chemotherapy			0.47
No	34 (35.05%)	63 (64.95%)	
Yes	39 (30.23%)	90 (69.77%)	
Previous radiation to local bone metastasis			>0.99
No	54 (32.14%)	114 (67.86%)	
Yes	19 (32.76%)	39 (67.24%)	
Previous radiation to extra-skeletal metastases			0.78
No	32 (33.33%)	64 (66.67%)	
Yes	41 (31.54%)	89 (68.46%)	
Surgical location(s)			0.38
Lower extremity	49 (30.06%)	114 (69.94%)	
Upper extremity	20 (36.36%)	35 (63.64%)	
Multiple limb surgery^∗∗^	4 (50.00%)	4 (50.00%)	
Impending versus pathologic fracture			**0.0064**
Impending fracture	22 (22.45%)	76 (77.55%)	
Pathologic fracture^a^	51 (39.84%)	77 (60.16%)	
Surgery performed			0.44
Intramedullary nail	40 (28.78%)	99 (71.22%)	
Arthroplasty	8 (34.78%)	15 (65.22%)	
Curettage, plate, cement	21 (37.50%)	35 (62.50%)	
Multiple limb surgery^∗∗^	4 (50.00%)	4 (50.00%)	
Mean surgery time in minutes (SD, Range)^∗^	86 (39.16, 36–258)	75 (31.12, 27–196)	**0.021**
Type of anesthesia			0.12
General	68 (31.19%)	150 (68.81%)	
Neuraxial	5 (62.50%)	3 (37.50%)	
Presurgery ambulatory status			0.34
Ambulatory	53 (30.29%)	122 (69.71%)	
Nonambulatory	10 (34.48%)	19 (65.52%)	
Unknown	10 (45.45%)	12 (54.55%)	
Smoking history			0.32
Nonsmoker	31 (28.97%)	76 (71.03%)	
Ever-smoker	42 (35.29%)	77 (64.71%)	
Mean smoking pack-year history	16.19 (18.48, 0–75)	14.56 (20.51, 0–100)	0.56
Cardiac disease			0.079
No	53 (29.44%)	127 (70.56%)	
Yes^a^	20 (43.48%)	26 (56.52%)	
Pulmonary disease			0.45
No	63 (33.51%)	125 (66.49%)	
Yes	10 (26.32%)	28 (73.68%)	
Vascular disease			0.53
No	68 (31.78%)	146 (68.22%)	
Yes	5 (41.67%)	7 (58.33%)	
Diabetes mellitus			0.58
No	62 (33.33%)	124 (66.67%)	
Yes	11 (27.50%)	29 (72.50%)	
Renal failure			0.59
No	69 (32.86%)	141 (67.14%)	
Yes	4 (25.00%)	12 (75.00%)	
Liver disease			>0.99
No	72 (32.29%)	151 (67.71%)	
Yes	1 (33.33%)	2 (66.67%)	
Nononcologic immunosuppressive disease			0.78
No	67 (31.90%)	143 (68.10%)	
Yes	6 (37.50%)	10 (62.50%)	
Blood transfusion			**0.03**
No	36 (26.67%)	99 (73.33%)	
Yes	37 (40.66%)	54 (59.34%)	
Mean blood product units transfused^a^	1.56 (2.15, 0–11)	1.02 (1.88, 0–14)	**0.045**

SD: standard deviation, ^∗^surgery time is measured from initial incision until final wound dressing is placed. ^∗∗^Multiple limb procedures in one surgery. ^a^Statistically significant risk factor for systemic complication. The bold values indicate statistically significant.

**Table 4:** Univariate analysis for 30-day mortality.

Variable	Frequency (%) or mean (SD, range)		*p* value
	Deceased (*n* = 39)	Alive (*n* = 187)	
Sex			0.86
Female	21 (17.95%)	96 (82.05%)	
Male	18 (16.51%)	91 (83.49%)	
Mean age at time of surgery	66.08 (10.76, 45–90)	67.61 (11.64, 19–91)	0.45
Mean time (in months) between index cancer diagnosis and surgery	28.59 (50.02, 0–174)	34.03 (52.78, 0–358)	0.55
Primary cancer			**0.048**
Multiple myeloma	3 (5.77%)	49 (94.23%)	
Lymphoma	2 (16.67%)	10 (83.33%)	
Lung	12 (27.91%)	31 (72.09%)	**0.046**
Breast	7 (15.56%)	38 (84.44%)	
Renal cell	2 (15.38%)	11 (84.62%)	
Prostate	3 (15.79%)	16 (84.21%)	
Other	10 (23.81%)	32 (76.19%)	
Metastatic burden			**0.022**
Bone only	6 (8.45%)	65 (91.55%)	
Bone and extra-skeletal	33 (21.29%)	122 (78.71%)	
Previous chemotherapy			>0.99
No	17 (17.53%)	80 (82.47%)	
Yes	22 (17.05%)	107 (82.95%)	
Previous radiation to local bone metastasis			0.84
No	30 (17.86%)	138 (82.14%)	
Yes	9 (15.52%)	49 (84.48%)	
Previous radiation to extra-skeletal metastases			0.6
No	15 (15.62%)	81 (84.38%)	
Yes	24 (18.46%)	106 (81.54%)	
Surgical location(s)			0.28
Lower extremity	28 (17.18%)	135 (82.82%)	
Upper extremity	8 (14.55%)	47 (85.45%)	
Multiple limb surgery^∗∗^	3 (37.50%)	5 (62.50%)	
Impending versus pathologic fracture			**0.0071**
Impending fracture	9 (9.18%)	89 (90.82%)	
Pathologic fracture	30 (23.44%)	98 (76.56%)	
Surgery performed			0.32
Intramedullary nail	24 (17.27%)	115 (82.73%)	
Arthroplasty	2 (8.70%)	21 (91.30%)	
Curettage, plate, cement	10 (17.86%)	46 (82.14%)	
Multiple limb surgery^∗∗^	3 (37.50%)	5 (62.50%)	
Mean surgery time in minutes (SD, Range)^∗^	85 (45.25, 37–258)	77 (31.49, 27–196)	0.19
Type of anesthesia			0.14
General	36 (16.51%)	182 (83.49%)	
Neuraxial	3 (37.50%)	5 (62.50%)	
Presurgery ambulatory status			0.28
Ambulatory	30 (17.14%)	145 (82.86%)	
Nonambulatory	3 (10.34%)	26 (89.66%)	
Unknown	6 (27.27%)	16 (72.73%)	
Smoking history			0.16
Nonsmoker	14 (13.08%)	93 (86.92%)	
Ever-smoker	25 (21.01%)	94 (78.99%)	
Mean smoking pack-year history	20.53 (18.08, 0–60)	14.89 (20.26, 0–100)	**0.033**
Cardiac disease			0.083
No	27 (15.00%)	153 (85.00%)	
Yes	12 (26.09%)	34 (73.91%)	
Pulmonary disease			0.1
No	36 (19.15%)	152 (80.85%)	
Yes	3 (7.89%)	35 (92.11%)	
Vascular disease			0.44
No	36 (16.82%)	178 (83.18%)	
Yes	3 (25.00%)	9 (75.00%)	
Diabetes mellitus			0.49
No	34 (18.28%)	152 (81.72%)	
Yes	5 (12.50%)	35 (87.50%)	
Renal failure			>0.99
No	37 (17.62%)	173 (82.38%)	
Yes	2 (12.50%)	14 (87.50%)	
Liver disease			>0.99
No	39 (17.49%)	184 (82.51%)	
Yes	0 (0%)	3 (100%)	
Nononcologic immunosuppressive disease			0.49
No	35 (16.67%)	175 (83.33%)	
Yes	4 (25.00%)	12 (75.00%)	
Blood transfusion			**0.031**
No	17 (12.59%)	118 (87.41%)	
Yes	22 (24.18%)	69 (75.82%)	
Mean blood product units transfused	1.95 (2.53, 0–11)	1.04 (1.81, 0–14)	**0.015**

SD: standard deviation, ^∗^surgery time is measured from initial incision until final wound dressing is placed. ^∗∗^Multiple limb procedures in one surgery. The bold values indicate statistically significant.

**Table 5:** Multivariate analysis for 30-day complications and mortality.

Variable	OR	95% CI	*p* value
*Complications*			
Metastatic burden			
Bone and extra-skeletal	2.12	1.14 to 4.37	**0.029**
Impending versus pathologic fracture			
Pathologic fracture	2.14	1.16 to 4.00	**0.016**
Mean surgery time	1.01	1.00 to 1.02	0.2
Blood transfusion			
Yes	1.35	0.56 to 3.20	0.5
Mean blood product units transfused	1.04	0.83 to 1.29	0.72
*Mortality*			
Primary cancer			
Lung	1.71	0.65 to 4.40	0.27
Metastatic burden			
Bone and extra-skeletal	4.14	1.56 to 13.44	**0.0085**
Impending versus pathologic fracture			
Pathologic fracture	2.84	1.27 to 6.86	**0.014**
Blood transfusion			
Yes	0.83	0.28 to 2.36	0.73
Mean blood product units transfused	1.27	1.00 to 1.62	**0.048**
Mean smoking pack-year history	1.00	0.98 to 1.02	0.89

OR: odds ratio, CI: confidence interval. The bold values indicate statistically significant.

## Data Availability

The data supporting the results of this study are available within the results' section and tables.
